# China’s low-carbon policy intensity dataset from national- to prefecture-level over 2007–2022

**DOI:** 10.1038/s41597-024-03033-5

**Published:** 2024-02-16

**Authors:** Xinyang Dong, Can Wang, Fang Zhang, Haowen Zhang, Chengqi Xia

**Affiliations:** 1 State Key Joint Laboratory of Environment Simulation and Pollution Control (SKLESPC), School of Environment, Tsinghua University, Beijing, 100084 China; 2 School of Public Policy and Management, Tsinghua University, Beijing, 100084 China

**Keywords:** Climate-change policy, Environmental economics

## Abstract

Low-carbon policies are essential for facilitating manufacturing industries’ low-carbon transformation and achieving carbon neutrality in China. However, recent studies usually apply proxy variables to quantify policies, while composite indices of policy intensity measured by objectives and instruments focus more on the national level. It is deficient in direct and comprehensive quantification for low-carbon policies. Hence, having extended the meaning of policy intensity, this paper constructs a low-carbon policy intensity index quantified by policy level, objective and instrument via phrase-oriented NLP algorithm and text-based prompt learning. This process is based on the low-carbon policy inventory we built for China’s manufacturing industries containing 7282 national-, provincial- and prefecture-level policies over 2007–2022. Lastly, we organize the dataset in two formats (.dta and .xlsx) for multidiscipline researchers. Apart from the inventory and intensity for each policy, the policy intensity is also aggregated to national-, provincial- and prefecture-level with sub-intensity for four objectives and three instruments. This dataset has potential uses for future studies by merging with macro and micro data related to low-carbon performances.

## Background & Summary

Under the goal of carbon neutrality, building green and low-carbon transformation systems and facilitating high-quality development of manufacturing industries are of great importance to achieving ambitious decarbonization targets^1^. Affected by the developing stage, China’s manufacturing industries are exposed to the dilemma of reducing carbon emissions and promoting economic development^2^, which requires governments’ support and stimulation through low-carbon policies. However, owing to the data limitation, what types of low-carbon policies are effective in facilitating low-carbon transformation and industrial upgrading are still unclear. Therefore, it is imperative to construct a comprehensive low-carbon policy intensity index based on policy texts from national- to prefecture-level.

Policy intensity is an index reflecting the stringency and importance of policy, which weighs through policy objectives and policy instruments^3–7^. While policy objectives mainly focus on issues that low-carbon policies formally aim to address, policy instruments pay attention to general norms that guide implementation preferences^4,7^. Based on this “policy objective–policy instrument” pattern, the meaning of policy intensity has been interpreted from different perspectives. Some studies coded objectives in terms of emission reduction and renewable energy production^8^. Other researchers divided policy instruments into demand-side, environmental and supply-side instruments^9^. Meanwhile, six measures containing objectives, scope, budget, implementation, and monitoring were also adopted to build policy intensity^8^.

However, a majority of policy intensity indices are constructed and discussed at the national level, ignoring the contribution of policy effects from sub-national regions. For example, an environmental policy stringency (EPS) index developed by OECD measured 13 policy instruments for 40 countries^10,11^. By investigating countries’ policy portfolios from 1998 to 2010, an index of climate policy activity was constructed for the energy production sector in Austria, Germany, and the United Kingdom^8^. To be more specific, policy level is an essential factor influencing the intensity of policy instruments. In general, national policies are more overarching, which provide developing directions and guidance for the whole country but promulgate few region-specific actions. Sub-national policies (e.g., provincial-level, prefecture-level instruments) are more focused and capable of devising appropriate measures suitable to local conditions. Hence, some policies from lower administration levels could have a higher intensity than their counterparts from higher policy levels, which needs to be quantified through policy intensity.

Moreover, studies pay less attention to the policy intensity of low-carbon policies. Previous literature mainly used dummy variables or ordinal variables to quantify the individual effect of pilot^12–15^, short-term^16^, regional and industrial^17^ low-carbon policies, which are not able to evaluate the effects of low-carbon policies as a whole. Meanwhile, when it comes to different types of low-carbon policy instruments, although effects of tax measures, subsidies, investment incentives, bidding systems, voluntary programs, quantity obligations, and environmental protection laws have been discussed by several researchers^18,19^, no consistent conclusion has been made for command-and-control regulation and market-based policies.

From the methodology perspective, owing to the immense progress made in machine learning and artificial intelligence, it is possible to construct policy indicators based on the meaning of policy texts and these methods, which could have limited human biases and increase the effectiveness of policy indices^20^. However, this research area has not been sufficiently developed, and only a few studies built low-carbon or environmental indicators based on machine learning. On the one hand, a semi-automated policy analysis tool with a labelling process and sentence-BERT (SBERT) model was used to identify forest policies and classify policy instruments^21^. On the other hand, China’s national-level environmental policy intensity for 1978–2019 has been constructed by policy text analysis and machine learning algorithms (e.g., random forest, support vector machine, Ridge), which shows the policy-intensity evolution towards environmental issues^5^.

Therefore, this paper aims to build a low-carbon policy intensity index from 2007 to 2022 by prompt learning for China’s manufacturing industries, which contributes to the literature in three aspects. First, the meaning of low-carbon policy intensity is deepened by adding the factor of policy level, which is based on 7282 low-carbon policies promulgated from the nation, 31 provinces and all 334 prefecture cities for manufacturing industries. Hence, following the “policy objective-policy instrument” pattern, the low-carbon policy intensity in this paper is quantified by multiplying each policy’s objective, instrument, and level. Second, the construction process of low-carbon policy intensity combines phrase-oriented NLP algorithm and text-based prompt learning, which is a few-shot learning suitable for policy texts with smaller samples and higher manual labelling costs. This new paradigm in NLP field avoids the need for large samples in pre-train and fine-tuning paradigms. Meanwhile, it also reduces human biases and the non-repeatability brought by traditional 100% manual policy scoring. Lastly, this study further aggregates sub-intensity indices for three policy levels (i.e., national-, provincial-, and prefecture-level), four policy objectives (i.e., carbon reduction, energy conservation, capacity utilization, and technology), and three policy instruments (i.e., command-and-control, market-based, composite instruments). It is more convenient for future research to merge this dataset with macro- and micro-data for extended analysis and discuss the impact of low-carbon policies from different perspectives.

## Methods

**Fig. 1:**
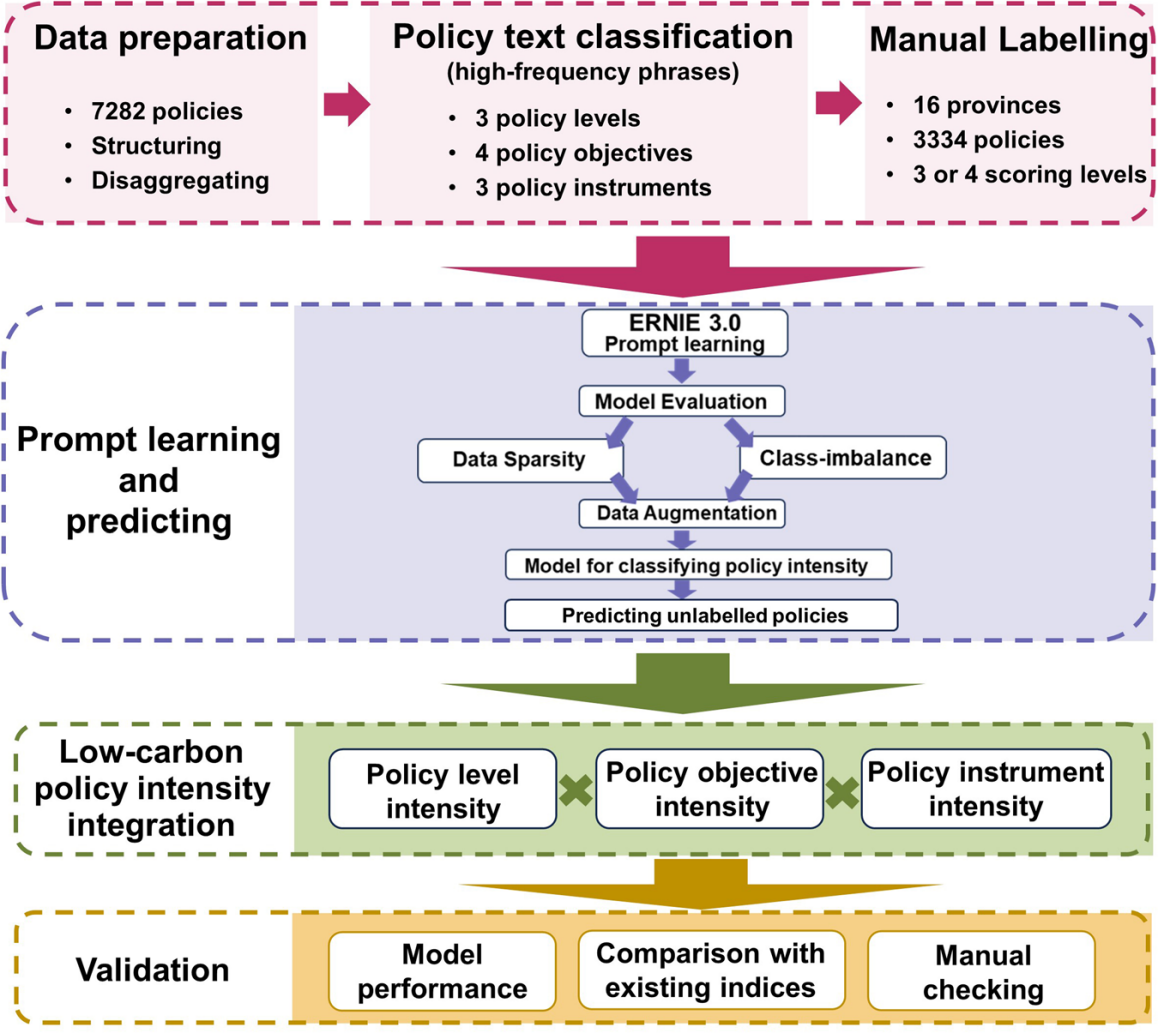
The research framework of building low-carbon policy intensity.

The research framework of this paper includes six modules, which are displayed in Fig. 1. Based on the constructed low-carbon policy inventory with 7282 policies over 2007–2022, this paper extends the “policy target-policy instrument” pattern and uses prompt learning to quantify the low-carbon policy intensity, thereby calculating the policy intensity via multiplying policy level, objective and instrument.

### Data preparation

The section for data preparation lays the foundation for building low-carbon policy intensity, which is achieved through three steps: constructing a low-carbon policy inventory, structuring policy texts, and disaggregating texts into policy objectives and policy instruments.

#### Step 1: Constructing a low-carbon policy inventory

This paper collects low-carbon policies from PKULaw.com (https://pkulaw.com/), which is a policy database containing not only national policies but also policies from provincial- and prefecture-level. This paper focuses on different administration levels’ low-carbon policies from 2007 to 2022. 2007 is of great importance for China’s climate change issue when a national leading group headed by the Premier of the State Council and with officials from 30 ministries and commissions as members was founded in response to climate change, energy conservation, and emission reduction. Since then, China’s low-carbon policy has gradually changed from being attached to various environmental policies to an important policy category with more independent policies.

When selecting low-carbon policies, this paper defines a generalized scope of “low-carbon”. Keywords such as “carbon reduction”, “greenhouse gases”, “energy conservation”, “energy efficiency”, “energy consumption”, and “overcapacity” have been adopted to search policies, since policies in these fields are able to directly or indirectly mitigate climate change and influence low carbon development. Meanwhile, we put more emphasis on targeted and implementable policies, which have relatively clear low-carbon policy objectives and instruments with carbon reduction potential. To be more specific, despite higher legal forces, laws and outlines for quality development related to low-carbon and climate change are excluded from the policy inventory. Meanwhile, policies related to publicity have not been considered, which are more likely to raise awareness rather than taking action.

Finally, this paper builds a low-carbon policy inventory with 7282 policy texts from national-, provincial- and prefecture-level by careful selection. Policies from central and local departments related to development and reform, ecology and environment, finance, industry and information technology, and taxation are included in the inventory. Table 1 displays departments at different administration levels that have promulgated low-carbon policies.

**Table 1:** Departments from different levels that have promulgated low-carbon policies.

National-level	Provincial-level	Prefecture-level
State Council	• Government of Province	Government of Municipality
	• Government of Autonomous Region	
	• Government of Municipality (For Beijing, Tianjin, Shanghai, and Chongqing only)	
National Development and Reform Commission	• Provincial Development and Reform Commission	Municipal Development and Reform Commission
	• Autonomous Region Development and Reform Commission	
	• Municipal Development and Reform Commission (For Beijing, Tianjin, Shanghai, and Chongqing only)	
Ministry of Ecology and Environment (Ministry of Environment Protection before 2018)	• Department of Ecology and Environment of Province	Municipal Ecology and Environment Bureau
	• Department of Ecology and Environment of Autonomous Region	
	• Municipal Ecology and Environment Bureau (For Beijing, Tianjin, Shanghai, and Chongqing only)	
Ministry of Finance	• Department of Finance of Province	Municipal Finance Bureau
	• Department of Finance of Autonomous Region	
	• Municipal Finance Bureau (For Beijing, Tianjin, Shanghai, and Chongqing only)	
Ministry of Industry and Information Technology	• Department of Industry and Information Technology of Province	Municipal Industrial and Information Technology Bureau
	• Department of Industry and Information Technology of Autonomous Region	
	• Municipal Industrial and Information Technology Bureau (For Beijing, Tianjin, Shanghai, and Chongqing only)	
State Taxation Administration	• Provincial Tax Service, State Taxation Administration	Municipal Tax Service, State Taxation Administration
	• Autonomous Region Tax Service, State Taxation Administration	
	• Municipal Tax Service, State Taxation Administration (For Beijing, Tianjin, Shanghai, and Chongqing only)	

The Ministry of Ecology and Environment was founded in 2018, while the Ministry of Environment Protection was partly responsible for launching low-carbon policies before 2018. Meanwhile, some local departments affiliated with the Ministry of Industry and Information Technology use the expression “Economic and Information Technology”.

#### Step 2: Structuring policy texts

It is known that policy texts have a high degree of fixed features, which contain structural information such as policy title, policy background, policy objectives, policy instruments, issuing institution, and publication year^8^. Hence, it is more convenient to grasp the meaning of whole policy texts by breaking it into different parts.

During the structuring process, headings and subheadings are key marks of each section. Headings of policy texts usually include sections for guiding ideology, working principle, target and goals, and supporting measures, which are led by phrases such as “Chapter X” and “Chinese number、”. Subheadings of policy texts are detailed sub-sections of headings, led by “(Chinese number)” or “Arabic figure、”. Thus, by locating lines with those special phases, headings, subheadings as well as contents for each part in the text file are able to be extracted respectively and sequentially to each cell of Excel in columns “title” and “content”.

#### Step 3: Disaggregating policy texts into the policy-objective file and policy-instrument file

Based on the Excel file generated in Step 2, we refine texts for policy objective and policy instrument into two separate files by keywords. On the one hand, “target”, “tasks”, “indices”, and “policy guidance” are keywords in headings, subheadings and contents for selecting policy objectives. Those cells are selected and sorted by subheadings, headings, and contents before being saved as a new text file for policy objective. Because the description of policy objectives in subsections tends to be more specified with clear expression than the counterparts. For some policy texts without headings or subheadings, sentences related to policy objectives cannot be located and have to be extracted from the whole content. On the other hand, policy instruments have diverse keywords and need to adopt an exclusion method. Hence, after dropping parts for policy objective and background (e.g., status, meaning, guidance, principle, general thoughts, requirements), the remaining parts of the policy are saved as a new text file for policy instrument.

### Policy text classification

Having followed the “policy objective-policy instrument” pattern and refined text files for policy objective and instrument^5,8^, we consider three factors influencing low-carbon policy intensity, namely, policy level, policy objective, and policy instrument. Based on the low-carbon policy inventory we built, this paper classifies policies into national-, provincial-, and prefecture-level, which are promulgated by departments of the nation, provinces, autonomous regions, or cities. As for policy objectives, we follow the scope of “low-carbon” defined in this paper, and include policy objectives for carbon reduction, energy conservation, capacity utilization, and technology. Carbon reduction is the composite objective directly related to low carbon, while energy conservation is the composite objective for energy consumption and efficiency indirectly associated with low carbon. The remaining two objectives focus on specific fields of easing overcapacity, technological innovation, and industrial upgrading, which show an indirect connection with low carbon. Finally, based on existing studies^5,22^, we not only include policies with single command-and-control or market-based instruments, but also define policies with composite instruments (i.e., having command-and-control and market-based instruments in one policy at the same time).

Apart from the policy level, policy objectives and instruments are classified through high-frequency phrases extracted from policy texts and titles. First, during the process of data preparation, a lexicon has been constructed for policy classification, containing keywords and detailed expressions for titles and contents. Following Tian *et al*.^23^, keywords related to different policy objectives and instruments are constructed by extensively checking through low-carbon literature, which include 38 seed words and are shown in Table 2. Furthermore, detailed expressions for titles and contents are extracted by using *word2vec* to acquire the semantic similarity between keywords and tokenized policy contents. To be more specific, having calculated the similarity between the word vectors of each phrase in policy contents and keywords, we selected 90 synonym words with the highest similarity to each keyword. After manually checking and excluding phrases with inappropriate meanings to low-carbon, a lexicon for policy classification is constructed and applied to calculate phrase frequency for policy text classification.

**Table 2:** Selection of keywords in different dimensions for policy text classification.

Policy dimension	Subdimension	Keywords	Sources
Policy objectives	Carbon reduction	Carbon emission	Zhao *et al*.^30^
		Carbon intensity	
		Climate change	
		GHG emission	
		Carbon peaking	
		Carbon neutrality	
		Low-carbon city pilot	Pan *et al*.^14^
		Emission trading pilot	Zhu *et al*.^12^
		Green finance	Su *et al*.^31^
		Ecological civilization	Zhang and Fu^32^
	Energy conservation	Energy conservation	Pardo Martínez and Silveira^33^
		Energy consumption	
		Energy intensity	
		Energy efficiency	
		Differential power pricing	Lin and Liu^34^
		Target responsibility system	Sun^35^
		Ten-Thousand Enterprises Program	Lo *et al*.^36^
	Capacity utilization	Overcapacity	Zhu *et al*.^37^
		Withdrawal of outdated capacity	
		Air pollution control	Zhao *et al*.^38^
	Technology	Industrial development	Du *et al*.^39^
		Technology innovation	
		R&D	
		Productivity	
Policy instruments	Command-and-control	Penalty	Turken *et al*.^40^
		Prohibition/Ban	Knill *et al*.^3^
		Permits	
		Standard	Tang *et al*.^16^
		Restricted use	
		Target responsibility system	Sun^35^
	Market-based	Subsidies	Zhang *et al*.^41^
		Taxes	
		Loan	
		Risk guarantees	Jonestone *et al*.^18^
		Grants	
		Tradable certificates	
		Price supports	
		Investment incentives/Finance	

Second, in the policy classification, one policy only belongs to a single category of policy objectives and policy instruments. For policies with the same number of phrase frequency in multiple categories, it is necessary to set the priority for classification. In this paper, composite objectives (i.e., carbon reduction, energy conservation) take precedence over specific fields (i.e., capacity utilization, technology), and direct policy objective (i.e., carbon reduction) is given priority over the indirect counterpart (i.e., energy conservation). For policy instruments, on the basis of using high-frequency phrases to divide command-and-control and market-based instruments, it is necessary to define the grouping criteria for composite instruments. The average phrase frequency for market-based instruments is 10 phrases higher than the counterpart for command-and-control instruments. Hence, policies are classified into the composite instrument if the phrase frequencies for both instruments are greater than 10 and the difference is within 10.

### Quantifying low-carbon policy intensity

Having classified policies into different categories, we add the factor of policy level into “policy objective-policy instrument” pattern and calculate the low-carbon policy intensity for each policy using Eq. (1):

1 $$P{I}_{r,a,t}={L}_{r,a,t,l}\times {O}_{r,a,t,o}\times {I}_{r,a,t,i}$$

where $P{I}_{r,a,t}$ is the low-carbon policy intensity for policy *a* in region *r* and year *t*. Region *r* could be a nation, a province *ρ* or a prefecture-level city *c*. ${L}_{r,a,t,l}$ is the intensity of policy level *l* for policy *a* in region *r* and year *t*. *O*_*r,a,t,o*_ is the intensity of policy objective *o* for policy *a* in region *r* and year *t*. ${I}_{r,a,t,i}$ is the intensity of policy instrument *i* for policy *a* in region *r* and year *t*.

**Fig. 2:**
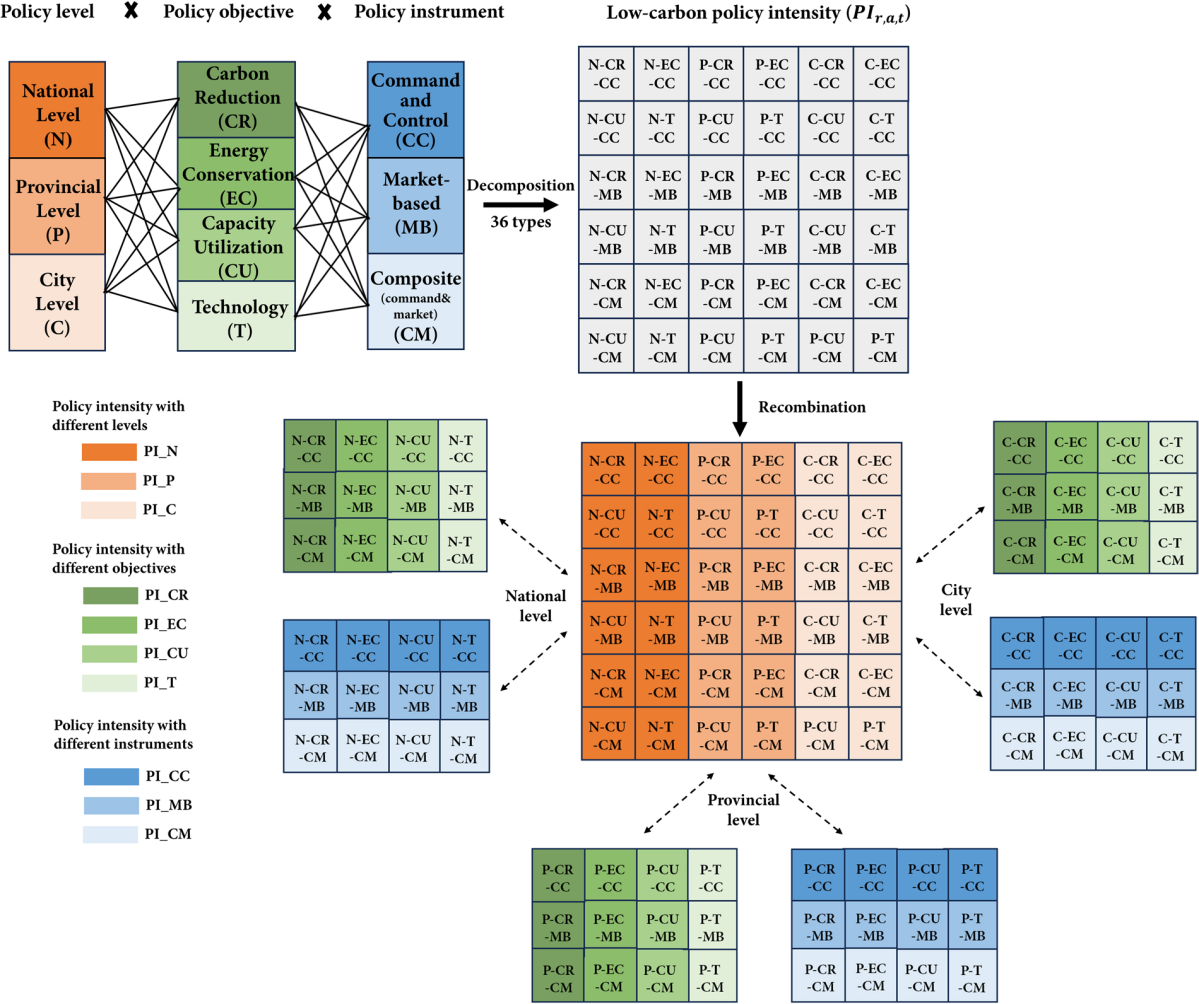
Process of aggregating low-carbon policy intensity.

Figure 2 shows the components of low-carbon policy intensity. To be more specific, the policy level *l* could be national-, provincial-, or prefecture-level. Policy objective *o* has four categories (i.e., carbon reduction, energy conservation, capacity utilization, technology). There are three types of policy instrument *i*, namely, command-and-control, market-based, and composite instruments.

As shown in Fig. 2, $P{I}_{r,a,t}$ includes 36 types of policies and can be aggregated in different patterns by Eqs. (2–4).

2 $$P{I}_{r,t}={\sum }_{a}P{I}_{r,a,t}$$

3 $$P{I}_{r,t}=PI\_C{R}_{r,t}+PI\_E{C}_{r,t}+PI\_C{U}_{r,t}+PI\_{T}_{r,t}$$

4 $$P{I}_{r,t}=PI\_C{C}_{r,t}+PI\_M{B}_{r,t}+PI\_C{M}_{r,t}$$

Calculated by Eq. (2), $P{I}_{r,t}$ is the sum of policy intensity promulgated by region *r* in year *t*. From the perspective of policy level, $P{I}_{r,t}$ could be $PI\_{N}_{t}$, $PI\_{P}_{\rho ,t}$ or $PI\_{C}_{c,t}$. $PI\_{C}_{c,t}$ is the sum of prefecture-level low-carbon intensity promulgated by prefecture-level city *c* in year *t*. $PI\_{P}_{\rho ,t}$ is the sum of provincial low-carbon intensity promulgated by province *ρ* in year *t*. $PI\_{N}_{t}$ is the national level low-carbon intensity in year *t*. In Eq. (3), $P{I}_{r,t}$ is summed by four different policy objectives. $PI\_C{R}_{r,t}$, $PI\_E{C}_{r,t}$, $PI\_C{U}_{r,t}$ and $PI\_{T}_{r,t}$ are policy intensities respectively aiming at carbon reduction, energy conservation, capacity utilization, and technology promulgated by region *r* in year *t*. Finally, $P{I}_{r,t}$ can also be aggregated by three different policy instruments in Eq. (4). $PI\_C{C}_{r,t}$, $PI\_M{B}_{r,t}$ and $PI\_C{M}_{r,t}$ are respectively policy intensities for command-and-control, market-based and composite instruments for region *r* in year *t*.

### Manual labelling

In order to create a supervised prompt learning model and predict intensities for policies’ objective and instrument, a manual annotation of 3334 policy texts in 16 provinces is used to train the model. The intensity of policy level ${L}_{r,a,t,l}$, policy objective *O*_*r,a,t,o*_, and policy instrument ${I}_{r,a,t,i}$ are respectively rated on a scale of 1 to 3 (Label 1: weak intensity, Label 2: medium intensity, Label 3: high intensity, Label 4: highest intensity (for energy conservation objective only)).

For policy objectives, quantitative policy objectives with specific industrial, regional, or year-by-year supporting goals will be given a higher score. On the contrary, if the expression of policy objective is vague without quantitative or clear targets, the intensity will be relatively low. Unlike the scoring system for carbon reduction, capacity utilization, and technology goals, the policy objective for energy conservation has a 4-level scoring system and will be normalized within 3 (i.e., 3, 2.25, 1.5, 0.75) after scoring. Because the maximum level of energy conservation objective has been intensified after 2015, which changed from controlling the intensity of energy consumption to restricting the total amount and intensity of energy consumption at the same time. Hence, the energy conservation objective with level 4 is mainly used for scoring policies with “dual control of energy consumption”.

Meanwhile, the scoring criteria for three policy instruments are quite different. For command-and-control instruments, Label 3 will be given to policies for shutting down enterprises, eliminating excess production capacity and the target responsibility system for local government officials, which is related to their promotion, awards and punishment. Label 2 is mainly for strictly prohibiting additional production capacity, which has less impact on existing capacity. The intensity for market-based instruments is related to the clarity of policies’ economic supports. For composite instruments, since it includes command-and-control and market-based instruments at the same time, this type of policy will be respectively scored on the basis of two instruments. The maximum value of two labels will be taken as the intensity of composite instrument.

Detailed criteria and labelling examples for each objective and instrument are presented in Tables 3, 4.

**Table 3:** Labelling criteria of the intensity for different policy objectives.

Label	Scoring criteria	Labelling examples for different policy objectives			
		Carbon reduction	Energy conservation	Capacity utilization	Technology
Label 4	• (For energy conservation only) Goals controlling the total amount and intensity of energy consumption at the same time.		• Total energy consumption will be controlled at 72 million tce. Energy consumption per unit of GDP will be reduced by 3.5% compared with 2010.		
Label 3	• Containing clear quantitative targets with a single type and relevant specific supporting targets (e.g., industrial, regional, year-by-year).	• By 2020, CO_2_ per unit of GDP will be reduced by 20.5% compared with 2015. Targets of reducing carbon emission intensity for each district will be 22% or 21%.	• From 2006 to 2010, the energy consumption per unit of GDP will be reduced by 20% compared with 2005 (i.e., 4% in 2006, 5% in 2007, 5% in 2008, 4% in 2009 and 4% in 2010).	• By the end of 2020, the city’s coking industry will be reduced by 3.25 million tons. 1.08, 0.30, and 1.87 million tons will be reduced by the end of 2019 and 2020.	• Funding for basic research will account for 13% of R&D funding. The number of invention patents owned by 10,000 people will reach 80.
Label 2	• Containing quantitative targets without specific supporting targets.	• By 2015, CO_2_ per unit of GDP will be reduced by 17% compared with 2010. The low-carbon industrial system will be basically formed.	• In 2009, it is necessary to achieve a 4% reduction in energy consumption per unit of GDP and a 5% reduction in water consumption per unit of GDP.	• Regarding de-capacity, steel production capacity will be reduced to less than 14 million tons.	• A number of critical breakthroughs for electronic components will be made, and 15 related companies’ revenue scales will exceed 10 billion yuan.
	• Containing timelines or roadmaps with few quantitative goals.	• From 2021 to 2025, CO_2_ will continue to maintain the optimal level in provincial regions. By 2030, the goal of carbon peaking will be achieved as scheduled.			
Label 1	• Only mentioning policy objectives with vague expressions.	• Carbon emissions will continue to decline, and significant progress will be made in carbon neutrality.	• The energy consumption per unit of regional GDP will be further decreased to adjust and optimize the industrial structure.	• On the basis of the task of resolving excess capacity in the city, more cement capacity will be reduced to remain the capacity utilization rate in a reasonable range.	• By 2023, superior products will be further enhanced, and the security supply level of industrial chains will be significantly improved.
		• The province’s CO_2_ per unit of GDP will be controlled within the national decomposition goals.			

The scoring system for carbon reduction, capacity utilization, and technology goals are respectively rated on a scale of 1 to 3, while the policy objective for energy conservation has a 4-level scoring system and has been normalized within 3 (i.e., 3, 2.25, 1.5, 0.75) after scoring.

**Table 4:** Labelling criteria of the intensity for different policy instruments.

Label	Low-carbon policy with command-and-control instruments		Low-carbon policy with market-based instruments	
	Scoring criteria	Examples	Scoring criteria	Examples
Label 3	• Target responsibility system; shut down; phase-out obsolete capacity.	• Assessing the realization of climate change and energy conservation goals, and using the results as important factors for rewards, punishments, selecting and appointing officials	• Detailed quantitative measures (e.g., the amount, supporting objects of funds, subsidies, or investments).	• In the next two years, a total of 30 million yuan from the Municipal Environmental Protection Bureau will be allocated. Among them, about 10 million yuan for two years will be given for the technical transformation and upgrading of demonstration and promotion vehicles and the construction of an operation data monitoring and management platform.
Label 2	• Strictly prohibiting additional production capacity; negative list system for market access; mergers and acquisitions; mandatory disclosure; relocation.	• Officials will not approve projects that expand production capacity and must not delegate approval authority. No additional production capacity and expansion of electrolytic aluminium projects will be approved in the next two years.	• Containing a few quantitative economics measures without specific objects.	• No more than 20% or no more than 3 million yuan of financial supports will be given to the equipment investment of supporting facilities.
Label 1	• Policy instruments with vague expressions.	• Promoting green transformation and upgrading industries. Strengthening the constraints of energy conservation and emission reduction systems. Controlling the expansion of total production capacity and reducing excess production capacity.	• Policy instruments with vague expressions.	• Establishing Industrial Development Fund. Implementing fiscal and taxation, finance, land, employee placement, and other support policies. Removing institutional barriers to cross-regional and cross-ownership mergers and acquisitions.

Policies from composite instruments will be respectively scored on the basis of command-and-control and market-based instruments. The maximum value of two labels will be taken as the intensity of composite instruments.

### Prompt learning and prediction

Based on the dataset of manual labelling, this paper trains models for each objective and instrument, and predicts the intensity by prompt learning, which is a few-shot learning suitable for policy texts with a smaller sample and higher manual labelling costs. Before prompt learning, the dominant pre-train and fine-tune paradigm in NLP requires a fixed architecture pre-trained as a language model (LM) and adapts the LM model to different downstream tasks by adding additional parameters and fine-tuning^24^. Hence, it is inefficient due to the need for parameters for every single project, and the gap between large-scale unsupervised learning and downstream tasks. Compared with the paradigm mentioned above, prompt learning is able to reformulate downstream tasks, makes it similar to the original pre-trained LM, and predicts desired output without additional training for tasks with the help of a textual prompt^24^.

To be more specific, prompt learning requires three steps to make a text input *x* to predict label output *y* through learning the parameters *θ* of model $P\left(y| x;\theta \right)$ in the task for text classification^24^. First, a template is designed by the prompting function $prompt\;x{\prime} ={f}_{{\rm{prompt}}}\left(x\right)$ with an input slot *x* and an answer text *z*, which will be mapped to *y*. Then, based on the pre-trained LM and small word subset *Z* for classification, the highest-scoring text $\widehat{z}$ is searched using Eq. (5), which could maximize the score of LM. Finally, the highest-scoring answer $\widehat{z}$ is used to predict the highest-scoring label output $\widehat{y}$.

5 $$\widehat{z}=\mathop{{\rm{search}}}\limits_{z\in Z}P\left({f}_{fill}\left(x{\prime} ,z\right);\theta \right)$$

Therefore, this paper applies ERNIE 3.0 to conduct the task of classifying the intensity of texts for policy objectives and instruments through prompt learning. ERNIE 3.0 (https://wenxin.baidu.com/wenxin/modelbasedetail/ernie3/) is a large-scale knowledge enhanced pre-training model for language understanding, which has better performance on few-shot learning^25^. Having trained the model with 3334 labelled policy texts, we conduct a model evaluation to evaluate the training result. Then two strategies of data augmentation (TrustAI) are used to deal with data sparsity and class-imbalance. Then, models with the best accuracy are selected and used to predict the intensity of policy objectives and instruments for the remaining unlabelled policy texts.

### Low-carbon policy intensity integration

**Fig. 3:**
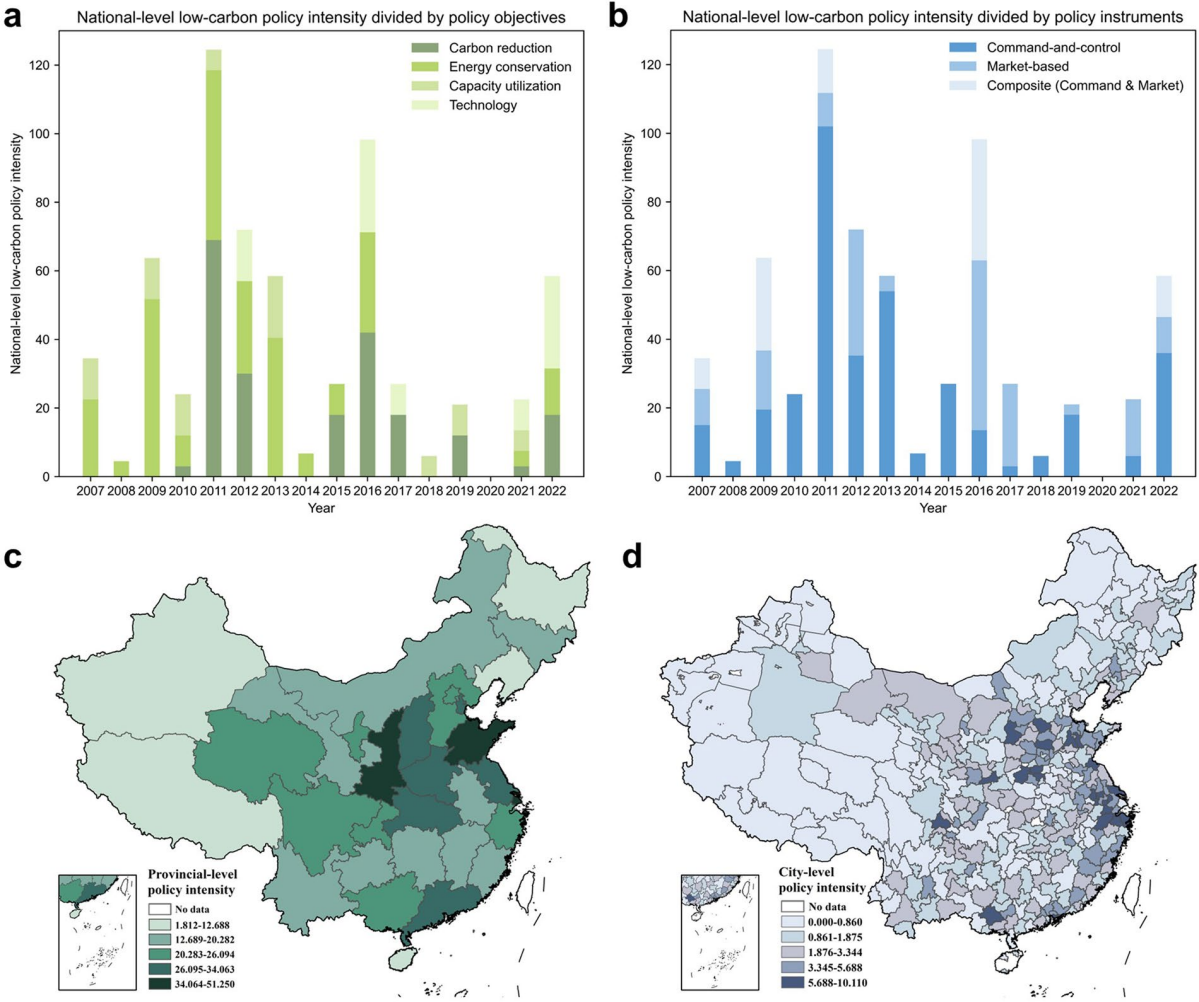
Heterogeneous low-carbon policy intensity from the perspective of policy level, time and, space. (**a**) and (**b**) show the national-level policy intensity divided by policy objectives and instruments. (**c,d**) are yearly average low-carbon policy intensity at the provincial- and prefecture-level.

After receiving all the intensity of policy objectives and instruments through manual labelling and prompt learning, the low-carbon policy intensity of each policy is multiplied by Eq. (1). Descriptive statistics of the low-carbon policy intensity are provided in the Supplementary Information. Figure 3 further shows the heterogeneity of low-carbon policy intensity from the perspective of policy level, time, and space. Figure 3a,b indicate strong cyclicality for national-level low-carbon policy intensity, which had stronger intensity in 2011 and 2016 (i.e., the first year of China’s 12^th^ and 13^th^ Five-Year Plan). Policies for energy conservation had a better continuity, while carbon reduction started to have independent policies in 2010 after China first proposed the carbon emission reduction target in 2009. Although command-and-control policies dominated the intensity of national low-carbon policies before 2015, market-based and composite instruments made greater contributions after 2015. The yearly average low-carbon policy intensity at the provincial- and prefecture-level in Fig. 3c,d show that prefecture-level policy intensity was relatively lower compared with the provincial-level counterpart. Shanghai (east), Shandong Province (east), and Shaanxi Province (northwest) had the largest average provincial low-carbon policy intensity. Among 334 prefecture-level cities, 21 cities’ prefecture-level policy intensities were in the highest level, including 11 cities in the east of China (e.g., Wuxi, Jiangsu Province), 3 cities in the north of China (e.g., Shijiazhuang, Hebei Province), 3 cities in the central of China (e.g., Luoyang, Henan Province), and other 4 cities from southwest, northwest, and south of China.

## Data Records

Not only the low-carbon policy inventory and intensity for each policy but also the aggregated low-carbon policy intensity for national-, provincial- and prefecture-level are organized in the dataset. We provide two different format datasets for multidiscipline researchers. The first format of the dataset is stored in Stata files (.dta), while the second format is in Excel files (.xlsx). Lexicons for policy classification and definitions of variables are also attached to the dataset. Detailed information for this dataset is listed in Table 5. These files are all accessible via figshare^26^.

**Table 5:** Detailed information for data files.

Folders	Contents for datasets
(Aggregated) Low-carbon policy intensity	Definitions of variables.xlsx
	Prefecture-level low-carbon policy intensity (.dta, .xlsx)
	Provincial level low-carbon policy intensity (.dta, .xlsx)
	National level low-carbon policy intensity (.dta, .xlsx)
	Total low-carbon policy intensity aggregated to prefecture-level (.dta, .xlsx)
	Total low-carbon policy intensity aggregated to provincial level (.dta, .xlsx)
(For each policy) Low-carbon policy inventory and intensity	Lexicons for policy classification.xlsx
	National-level low-carbon policy inventory and intensity (.dta, .xlsx)
	Provincial-level low-carbon policy inventory and intensity (.dta, 31 files)
	Provincial-level low-carbon policy inventory and intensity (.xlsx, 31 files)
	Prefecture-level low-carbon policy inventory and intensity (.dta, 27 files)
	Prefecture-level low-carbon policy inventory and intensity (.xlsx, 27 files)

The link of each policy has been provided in folder “(For each policy) low-carbon policy inventory and intensity” of the dataset, which could locate to policy texts in the Laws & Regulations of PKULAW Database. Full policy texts in this database are only available for subscribers. Hence, researchers need to make sure that the academic institutions or individuals have subscribed to this database if you want to reproduce the process based on this paper.

## Technical Validation

### Model performance

In this paper, we use prompt learning to train models and predict the intensity for each objective and instrument. Among 7282 policies in our low-carbon policy inventory, 3334 policies with labels are used to train and evaluate the prompt learning model. 80% of the labelled dataset is used for training, while the remaining 20% is for validation. Two data augmentation strategies (i.e., sparse data identification and training data augmentation) are applied to improve prompt learning models by dealing with data sparsity and class-imbalance. Table 6 summarizes the accuracy of each model, which reflects the ratio of how many predicted labels are equal to actual labels in the sample. A detailed explanation of accuracy is provided in the Supplementary Information. Table 6 shows that the policy objective for carbon reduction has the best performance through “Prompt learning + Sparse data identification”, while “Prompt learning + Training data augmentation” is more suitable for the remaining three policy objectives and two policy instruments.

**Table 6:** Accuracy of prompt learning models.

Type of model	Low-carbon policy objectives				Low-carbon policy instruments	
	Carbon reduction	Energy conservation	Capacity utilization	Technology	Command-and-control	Market-based
Prompt learning	0.78	0.79	0.84	0.66	0.77	0.90
Prompt learning + Sparse data identification	0.85	0.81	0.80	0.66	0.77	0.88
Prompt learning + Training data augmentation	0.75	0.87	0.87	0.82	0.82	0.92

### Comparison with existing studies

Due to the absence of a publicly available dataset for low-carbon policy intensity with three administrative levels, we cannot directly compare with existing datasets. However, there are present studies constructing environmental policy indices for China. On the basis of the “policy objectives-policy measures” pattern, Zhang *et al*. constructed China’s national-level environmental policy intensity for 1978–2019 by policy text analysis and machine learning algorithms^5^. OECD built a country-specific and internationally comparable environmental policy stringency index (EPS) on the basis of second-hand data for 13 policy instruments, which covers 40 countries from 1990 to 2020^10,11^. These two policy indices have subcategories or sub-indices (e.g., energy conservation and emission reduction, industrial upgrading, market-based policies, non-market-based policies, technology support), which are closely related to low-carbon policies. Hence, the trend similarity among policy indices mentioned above could be used to validate the low-carbon policy intensity index constructed in this paper.

**Fig. 4:**
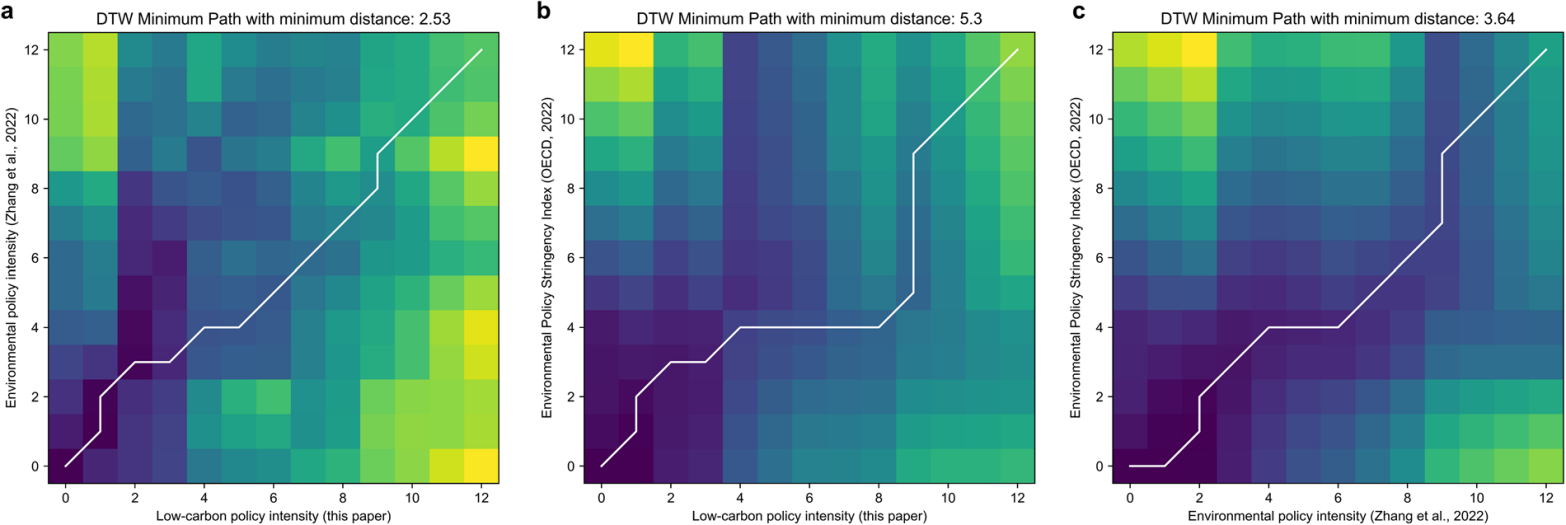
Path diagram with DTW distance between our national-level low-carbon policy intensity and existing studies. The horizontal axis of (**a,b**) are national-level low-carbon policy intensity in this paper. The vertical axis in (**a**) and (**b**) respectively represent environmental policy intensity from Zhang *et al*. and environmental policy stringency index from OECD. (**c**) compares indices between Zhang *et al*. and OECD.

Following Zhang *et al*., we apply dynamic time warping (DTW) to measure the similarity between two series that vary in trend. Having normalized three indices, three pairs of DTW distance are calculated by Euclidean distance, and paths minimizing DTW distance between three national indices are shown in Fig. 4. The distance between this paper and Zhang *et al*. is 2.53, being the smallest distance among three pairs. Its DTW minimum paths in the white line are small and almost parallel with the main diagonal. This shows that there is a highest degree of trend similarity between national-level indices of this paper and Zhang *et al*.’s environmental policy intensity, both of which are based on policy text analysis but with different training methodologies. That is to say, the low-carbon policy intensity built in this paper is able to reflect the changing trend of policy intensity.

### Manual checking

To further verify the accuracy of prompt learning prediction, we randomly select 500 policies through stratified random sampling based on policy objectives. Hence, each type of policy objective contains 125 policies to be manually labelled. From the perspective of policy instruments, these policies include 169 command-and-control policies, 276 market-based policies and 55 composite policies. Because composite policies are scored on the basis of two instruments, 224 command-and-control policies and 331 market-based policies need to be manually labelled. Table 7 presents the accuracy between results of prompt learning prediction and manual labelling, ranging from 0.85 to 0.93.

**Table 7:** Manual checking for prompt learning results.

Prompt learning VS Manual labelling	Low-carbon policy objectives				Low-carbon policy instruments	
	Carbon reduction	Energy conservation	Capacity utilization	Technology	Command-and-control	Market-based
True prediction	108	111	112	106	193	308
Total number	125	125	125	125	224	331
Accuracy	0.86	0.89	0.90	0.85	0.86	0.93

## Usage Notes

### Usage of low-carbon policy intensity

For the usage of this policy intensity index, the understanding of policy intensity needs to change from “policy-maker” to “policy-taker”. Hence, the meaning of $PI\_al{l}_{r,t}$ is the total low-carbon policy intensity received by region *r* in year *t*, which are $PI\_al{l}_{c,t}$ for city *c* in year *t* and $PI\_al{l}_{\rho ,t}$ for province *ρ* in year *t*. It is known that China is a centralized nation with the central government at the top and provincial, prefecture governments below it^27,28^. Under the planning- and goal-based governance, the central government usually disaggregates low-carbon targets among provinces, while provincial governments assign targets to prefecture-level cities through agreements and performance measures to achieve their targets. Hence, China has a multi-layered policy structure^29^, and governments not only implement policies from their own levels but also receive policies from higher administrative levels. To be more specific, when the research object is the performance of prefecture-level cities or firms, the received total low-carbon policy intensity $PI\_al{l}_{c,t}$ in Eq. (6) contains not only the intensity of prefecture-level policies but also those above-city policies (i.e., national- and provincial-level policies from which province it affiliates). If the study focuses on the provincial level, the total policy intensity $PI\_al{l}_{\rho ,t}$ in Eq. (7) received by provincial policy-takers includes both national and provincial policies.

Hence, the impact of policy intensity on city-level or firm-level policy-takers needs to be based on $PI\_al{l}_{c,t}$. If studies want to further discuss the impact of the policy intensity of different policy levels on carbon performances, the city-level intensity ($PI\_{C}_{c,t}$) and the above-city intensity $PI\_aboveCit{y}_{c,t}\left(=PI\_{P}_{\rho ,t}+PI\_{N}_{t}\right)$ in Eq. (6) can be added to the regression at the same time.

6 $$\begin{array}{ccc}PI\_al{l}_{c,t} & = & PI\_{C}_{c,t}+PI\_aboveCit{y}_{c,t}\\  & = & PI\_{C}_{c,t}+PI\_{P}_{\rho ,t}+PI\_{N}_{t}\end{array}$$

7 $$PI\_al{l}_{\rho ,t}=PI\_{P}_{\rho ,t}+PI\_{N}_{t}$$

### The starting and ending years of each policy

For user convenience, this dataset provides policies’ starting and ending years. Unlike the certain starting year of each policy, ending years of most policies in China are not clearly stated. Hence, policies’ end years have been verified in four criteria. First, the ending year of a few policies has been mentioned in the policy text (e.g., the planning period is 2012–2015, valid for 5 years, valid until 2021). Then, some policies have been abolished or replaced by new policies. Moreover, for policies without a clear ending year or abolishment, the policy end-year is verified on the basis of policy objectives. For example, although a policy was released in 2007 and hasn’t been abolished yet, expressions such as “at the end of 11th five-year plan period” and “until 2010” could be seen in the section of policy objective. Hence, the ending year of this policy is verified to be 2010. Lastly, for policies that cannot be confirmed using criteria mentioned above, the ending years of those policies are marked as “-” for uncertainty.

### Limitations and future work

This study has some possible limitations. On the one hand, limited by the availability of policy texts, this paper constructs the low-carbon policy intensity primarily based on policies that are publicly available. Hence, the intensity of those policy texts that have not been publicly released or only partly released in public cannot be fully evaluated. On the other hand, the release of policy texts and the execution from relevant departments are key factors affecting low-carbon policy effects. This paper mainly focuses on policy texts from the perspective of policy release, but pays less attention to policy execution. Future studies can further investigate the gap between the intensity of released low-carbon policy texts and strength of execution from different policy levels, which are likely to influence the progress of carbon reduction and low-carbon transformation.

### Supplementary information

Supplementary Information

## Data Availability

Code used for constructing the low-carbon policy intensity is written in Python 3.10.8 and Stata 15, and has been uploaded to figshare^26^.
