# Supplementary material for: China’s low-carbon policy intensity dataset from national- to prefecture-level over 2007–2022
Source: Sci Data. 2024 Feb 16;11:213. doi: 10.1038/s41597-024-03033-5 (PMC10873300; doi:10.1038/s41597-024-03033-5)
Supplement: Supplementary file 1 — Supplementary Information [file 41597_2024_3033_MOESM1_ESM.docx]

**Supplementary Information for**

**“China’s low-carbon policy intensity dataset from national- to prefecture-level over 2007-2022”**

Xinyang Dong^1^, Can Wang^1*^, Fang Zhang^2^, Haowen Zhang^1^, Chengqi Xia^1^

^1.^ State Key Joint Laboratory of Environment Simulation and Pollution Control (SKLESPC), School of Environment, Tsinghua University, Beijing, 100084, China.

^2.^ School of Public Policy and Management, Tsinghua University, Beijing, 100084, China.

^*^Corresponding author: Can Wang (canwang@tsinghua.edu.cn)

**Contents:**

Table S1. Descriptive statistics and variable definitions for low-carbon policy intensity

Note S1: The definition of accuracy in prompt learning

Fig. S1 Matrix between actual and predicted label for prompt learning

| **Variables** | **Definition** | **N** | **Mean** | **Std.Dev.** | **Min** | **Max** |
| --- | --- | --- | --- | --- | --- | --- |
| **Panel A: Prefecture-level low-carbon policy intensity** | | | | | | |
| $PI\_C$ | Prefecture-level PI | 5408 | 2.108 | 3.686 | 0.000 | 46.750 |
| $PI\_C\_CR$ | Prefecture-level carbon reduction PI | 5408 | 0.301 | 1.091 | 0.000 | 13.000 |
| $PI\_C\_EC$ | Prefecture-level energy conservation PI | 5408 | 1.119 | 2.283 | 0.000 | 24.750 |
| $PI\_C\_CU$ | Prefecture-level capacity utilization PI | 5408 | 0.339 | 1.437 | 0.000 | 18.000 |
| $PI\_C\_T$ | Prefecture-level technology PI | 5408 | 0.349 | 1.371 | 0.000 | 26.000 |
| $PI\_C\_CC$ | Prefecture-level command-and-control PI | 5408 | 0.924 | 2.249 | 0.000 | 34.250 |
| $PI\_C\_MB$ | Prefecture-level market-based PI | 5408 | 0.915 | 2.065 | 0.000 | 29.500 |
| $PI\_C\_CM$ | Prefecture-level composite PI | 5408 | 0.269 | 1.055 | 0.000 | 18.000 |
| **Panel B: Provincial level low-carbon policy intensity** | | | | | | |
| $PI\_P$ | Provincial level PI | 496 | 22.722 | 18.044 | 0.000 | 93.500 |
| $PI\_P\_CR$ | Provincial carbon reduction PI | 496 | 3.996 | 6.088 | 0.000 | 46.000 |
| $PI\_P\_EC$ | Provincial energy conservation PI | 496 | 11.044 | 11.217 | 0.000 | 75.000 |
| $PI\_P\_CU$ | Provincial capacity utilization PI | 496 | 3.972 | 6.967 | 0.000 | 50.000 |
| $PI\_P\_T$ | Provincial technology PI | 496 | 3.710 | 6.037 | 0.000 | 34.000 |
| $PI\_P\_CC$ | Provincial command-and-control PI | 496 | 8.262 | 9.383 | 0.000 | 63.500 |
| $PI\_P\_MB$ | Provincial market-based PI | 496 | 10.977 | 11.286 | 0.000 | 60.000 |
| $PI\_P\_CM$ | Provincial composite PI | 496 | 3.483 | 5.322 | 0.000 | 30.000 |
| **Panel C: National level low-carbon policy intensity** | | | | | | |
| $PI\_N$ | National level PI | 16 | 40.547 | 35.791 | 0.000 | 124.500 |
| $PI\_N\_CR$ | National carbon reduction PI | 16 | 13.313 | 19.534 | 0.000 | 69.000 |
| $PI\_N\_EC$ | National energy conservation PI | 16 | 16.734 | 17.905 | 0.000 | 51.750 |
| $PI\_N\_CU$ | National capacity utilization PI | 16 | 5.063 | 5.972 | 0.000 | 18.000 |
| $PI\_N\_T$ | National technology PI | 16 | 5.438 | 9.564 | 0.000 | 27.000 |
| $PI\_N\_CC$ | National command-and-control PI | 16 | 23.156 | 25.616 | 0.000 | 102.000 |
| $PI\_N\_MB$ | National market-based PI | 16 | 11.391 | 14.641 | 0.000 | 49.500 |
| $PI\_N\_CM$ | National composite PI | 16 | 6.000 | 10.903 | 0.000 | 35.250 |
| **Panel D: Total low-carbon policy intensity aggregated to prefecture-level** | | | | | | |
| $PI\_all\_C$ | Total PI received by prefecture-level | 5408 | 65.082 | 41.737 | 0.000 | 195.250 |
| $PI\_all\_C\_CR$ | Total carbon reduction PI received by prefecture-level | 5408 | 17.395 | 20.671 | 0.000 | 91.000 |
| $PI\_all\_C\_EC$ | Total energy conservation PI received by prefecture-level | 5408 | 28.240 | 21.359 | 0.000 | 100.500 |
| $PI\_all\_C\_CU$ | Total capacity utilization PI received by prefecture-level | 5408 | 9.587 | 9.109 | 0.000 | 63.000 |
| $PI\_all\_C\_T$ | Total technology PI received by prefecture-level | 5408 | 9.860 | 11.714 | 0.000 | 58.000 |
| $PI\_all\_C\_CC$ | Total command-and-control PI received by prefecture-level | 5408 | 32.255 | 27.002 | 0.000 | 144.750 |
| $PI\_all\_C\_MB$ | Total market-based PI received by prefecture-level | 5408 | 23.000 | 19.695 | 0.000 | 108.000 |
| $PI\_all\_C\_CM$ | Total composite PI received by prefecture-level | 5408 | 9.827 | 12.034 | 0.000 | 58.000 |
| **Panel E: Total low-carbon policy intensity aggregated to provincial level** | | | | | | |
| $PI\_all\_P$ | Total PI received by provincial level | 496 | 63.269 | 41.940 | 0.000 | 186.000 |
| $PI\_all\_P\_CR$ | Total carbon reduction PI received by provincial level | 496 | 17.308 | 20.722 | 0.000 | 89.000 |
| $PI\_all\_P\_EC$ | Total energy conservation PI received by provincial level | 496 | 27.779 | 21.588 | 0.000 | 91.500 |
| $PI\_all\_P\_CU$ | Total capacity utilization PI received by provincial level | 496 | 9.034 | 8.950 | 0.000 | 62.000 |
| $PI\_all\_P\_T$ | Total technology PI received by provincial level | 496 | 9.147 | 11.587 | 0.000 | 51.000 |
| $PI\_all\_P\_CC$ | Total command-and-control PI received by provincial level | 496 | 31.418 | 26.836 | 0.000 | 136.500 |
| $PI\_all\_P\_MB$ | Total market-based PI received by provincial level | 496 | 22.367 | 20.140 | 0.000 | 108.000 |
| $PI\_all\_P\_CM$ | Total composite PI received by provincial level | 496 | 9.483 | 11.882 | 0.000 | 53.500 |

**Table S1.** Descriptive statistics and variable definitions for low-carbon policy intensity

**Note S1: The definition of accuracy in prompt learning**

The accuracy of prompt learning reflects the ratio of how many predicted labels equal to actual labels in the sample. Based on the source code of paddlepaddle (<https://github.com/PaddlePaddle/Paddle/blob/release/2.5/python/paddle/metric/metrics.py>), the accuracy of prompt learning quantifies the overall correctness of the model’s prediction, which means actual labels equal to predicted labels on the main diagonal of the matrix in Fig. S1. That is to say, accuracy equals to equation (S1) for policy objectives or instruments with 3 labels in Fig. S1(a),

$\text{Accuracy}\text{=}\frac{A_{33}+A_{22}+A_{11}}{N_{3}+N_{2}+N_{1}}$ (S1)

while accuracy equals to equation (S2) for policies with energy conservation objective, which has 4-level labels in Supplementary Fig. S1(b):

$\text{Accuracy}\text{=}\frac{{A_{44}+A}_{33}+A_{22}+A_{11}}{N_{4}+N_{3}+N_{2}+N_{1}}$ (S2)

*
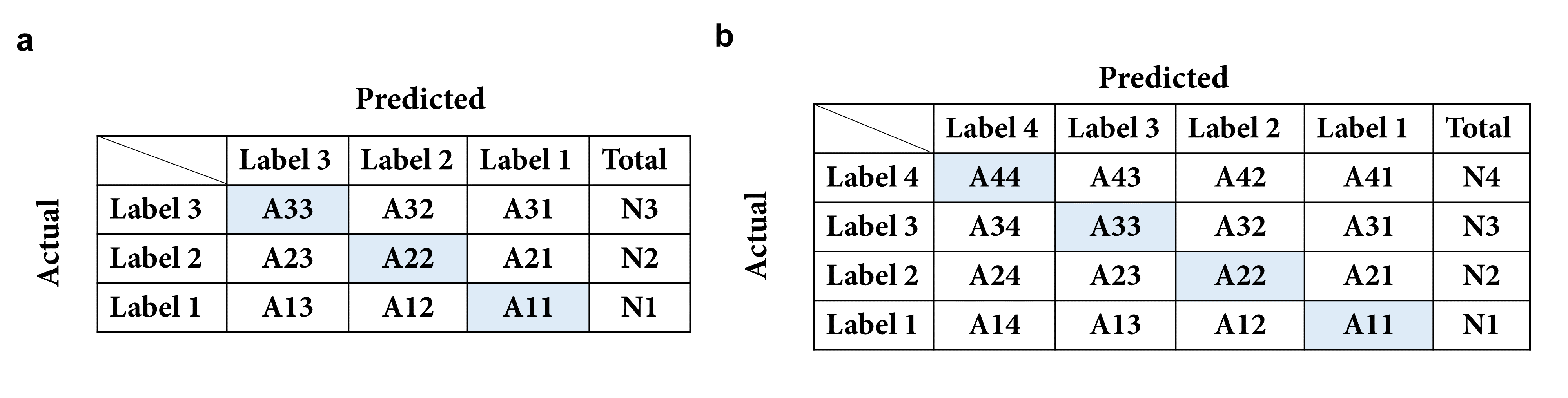
*

Fig. S1 Matrix between actual and predicted label for prompt learning
